# Phosphate Metabolism Modulation in Chronic Kidney Disease: When, How and to What Extent?

**DOI:** 10.5812/numonthly.18379

**Published:** 2014-04-29

**Authors:** Antonio Bellasi, Biagio Raffaele Di Iorio

**Affiliations:** 1 Division of Nephrology, Sant’ Anna Hospital, Como, Italy; 2 Department of Health Sciences, University of Milan, Italy; 3 Division of Nephrology, A. Landolfi Hospital, Solofra, Italy

**Keywords:** Phosphate, Metabolism, Bronchitis, Chronic, Pregnancy Outcome

Numerous observations have repeatedly shown that serum phosphorous is associated with poor survival (1). In a large cohort of 1,716 chronic kidney disease (CKD) referred to a Nephrology Center in Emilia Romagna, Italy (2), we investigated the robustness of the association between serum levels of phosphate and the risk of death by any cause or dialysis inception (2). Overall, we analyzed men (66% male) and women of 70 years of age [mean (standard deviation): age 70 (13.6) years] with CKD stage 4 [median estimated glomerular filtration rate (eGFR): 25 mL/min/1.73m^2^] (2). Over a relatively short period of time [1.3 (0.9) years] we recorded a total of 451 events (composite endpoint event rate: 202/1000 patient-year) equally distributed between the 2 outcome of interest (i.e. all-cause mortality and dialysis inception) (2). The risk of the composite endpoint was almost linearly and independently associated with serum phosphate levels. In this study cohort, hyperphosphatemia (serum phosphorous greater than 4.3 mg/dL) was observed in 25% of the study cohort and associated with a significant 104% (hazard ratio: 2.04; 95% Condfidence Interval: 1.44-2.90; P < 0.001) increased risk of either death or dialysis inception (2). Notably, in this study cohort, the risk associated with hyperphosphatemia was modulated by the presence of diabetes. Indeed, diabetic patients with hyperphosphaemia were at greater risk of unfavorable outcome when compared to non-diabetic patients with hyperphosphatemia (P for interaction test = 0.02) (2).

Though numerous studies corroborate these findings, several aspects of phosphorous balance and metabolism still need to be elucidated. What we measure in serum is only a small portion of the total body pool and is tightly regulated by a series of different factors (3). Diet intake, bone metabolism as well as kidney and intestinal excretion contribute to determine serum phosphorous levels however mechanism(s) that regulates phosphate excretion and exchange among body compartments are far from being elucidated (3). Studies that have investigated when hyperphosphatemia ensues in the course of CKD suggest that this condition may become prevalent when the GFR is reduced below 30 mL/min (i.e. CKD stage 4 according to the National Kidney Founfation classification) (4). However, phosphaturic factors such as fibrobast growth factor 23 (FGF23) or parathyroid hormone (PTH) are significantly increased at earlier stage of CKD and may compensate for a positive phosphate balance and prevent from hyperphosphatemia (5, 6). Thus, two different phases in the course of CKD can be distinguished: the first phase is characterized by a substantially preserved renal excretory capacity of phosphate and normal serum phosphate levels while the second is characterized by an insufficient renal excretion of phosphate and hyperphosphatemia (5). According to this view, hyperphosphatemia should be regarded as a sign of phosphate metabolism imbalance while normophosphatemia cannot discriminate neutral from positive and potentially dangerous phosphate balance. In a recent report by Dominguez et al. (7), 872 individuals recruited in the Heart and Soul Cohort with normal to mild renal function impairment and normal serum phosphorous were stratified according to serum levels of FGF23 and urinary levels of phosphate [i.e. fractional excretion of phosphate (FePi)] into 4 different groups (7). Notably, there was no clinically meaningful difference in serum phosphorous levels among the 4 groups (7). As documented in numerous other observational studies, FGF23 predicted the occurrence of any lethal as well as major CV events (7). However, high FePi substantially mitigated the risk associated with high levels of FGF23 (7), suggesting that an adequate phosphaturic response to FGF 23 reduces the risk associated with elevated FGF 23 and positive phosphate balance. These last findings corroborate the notion that FGF 23 is an adaptive system that in some circumstances, such as renal function decline, may turn into a maladaptive mechanism and contribute to the organ damage (8). Future studies should shed light on what are the factors involved in phosphate and mineral metabolism control and at what point in the course of CKD these metabolic abnormalities become harmful. Future effort is also needed to validate biomarkers that accurately reflect phosphate balance and to test whether a stringent control of the phosphorous body pool improves hard outcome in CKD patients.

Recent RCTs on phosphate management in CKD patients apparently yielded conflicting results (9, 10). In a small RCT of 149 CKD patients (estimated GFR = 20–45 mL/min per 1.73 m^2^) with normal serum phosphorous at study entry (mean serum phosphate: 4.2 mg/dL), investigators aimed at determining the effects of maximal dosage of commercially available phosphate binders (sevelamer carbonate, lanthanum carbonate and calcium acetate) on parameters of mineral metabolism (9). Albeit statistical significant, at study completion (9 months follow-up) authors could only detect a modest reduction in serum phosphate (from 4.2 to 3.9 mg/dL) in the active arm (9). A more pronounced effect was noticed when urinary excretion of phosphorous was considered (phosphate binders, but not placebo, decreased mean 24-hour urine phosphorus by 22%) suggesting that phosphatemia does not reflect accurately changes in phosphate balance in CKD patients with normal levels of serum phosphate (9). Of importance, active treatment was associated with a significant increase in coronary and abdominal aorta calcification in this study cohort suggesting some caution in the use of phosphate binders in CKD patients with normophosphatemia (9).

A more recent RCT (10), failed to demonstrate an effect of 9 months treatment with sevelamer carbonate vs. placebo on left ventricular mass (LVM) and pulse wave velocity (PWV) in a cohort of 109 CKD stage 3 subjects with normal serum phosphorous (mean serum phosphate 3.1 mg/dL) levels or signs of Chronic Kidney Disease Mineral Bone Disorder (CKD-MBD) at study inception (10). Though this study did not raise any safety concern (10), it does question the use of phosphate binders in normophosphatemic CKD patients. However, in light of the low baseline levels of FGF23 as well as the normal LVM of recruited individuals (10), it is likely that Chue and coworkers randomized patient in neutral phosphate balance and at low cardiovascular (CV) risk. These subjects may not benefit from a phosphate management intervention. Though we still await the RCT that demonstrates that lowering serum phosphorous level is beneficial, data is accumulating on the potential impact of different phosphate binder regimens on hard and surrogate outcome. In a pivotal study of 100 CKD stage 4 patients with hyperphosphatemia (> 6.0 mg/dL) (11), it was demonstrated that Sevelamer but not calcium acetate was able to reduce FGF23 and improve endothelial function (11). Though phosphorous control was different between study arms (changes in serum phosphorous: 7.7 to 5.3 and 7.7 to 6.5 mg/dL at baseline and study completion in the Sevelamer and calcium acetate, respectively), these results support the notion that calcium loads may contribute to FGF23 release and potentially prevent flow mediated dilatation restoration (11). Indeed, in an animal model of parathyrodectomized rats it was shown that FGF23 correlates with serum levels of calcium and high dietary calcium was associated with an increase in FGF23 (12). We investigated the impact of 2 different phosphate binder regimens on hard outcome (i.e. all-cause mortality and dialysis inception) and coronary artery calcification (CAC) progression in a cohort of 212 CKD stage 3-4 patients with high levels of serum phosphate (mean serum phosphate 4.84 mg/dL) (13). All patients were followed up to 36 months or until the occurrence of any lethal event or dialysis inception. At study completion, we documented a lower mortality and dialysis inception rate as well as a lower CAC progression among patients allocated to Sevelamer (13). Notably, the hypothesis that calcium-free vs. calcium-based phosphate binder may have a different impact on hard outcome is further corroborated by the results of 2 other RCTs (14, 15) and by a recent meta-analyses (16).

In summary, how to reconcile available evidence? A large and growing body of evidence suggests that phosphorous metabolism abnormalities are associated with adverse outcomes although we still need a RCT that demonstrates that phosphorous metabolism manipulation improves survival in CKD patients. Data suggest that serum phosphate is not an accurate marker of phosphate balance and it maybe useless for risk stratification especially in CKD patients with normophosphatemia. Future effort is needed to validate marker(s) of phosphate balance and to test whether these markers increase risk stratification accuracy and may guide treatment decision to improve outcome at the patient level. In line with the Kidney Disease: Improving Global Outcomes (KDIGO) clinical practice guideline on the management of chronic kidney disease-mineral and bone disorder (CKD-MBD) (17), recent RCTs suggest that the use of phosphate binders in patients with normal levels of serum phosphate maybe useless or even detrimental, especially if associated with a substantial calcium load. Lastly, data on comparison of calcium free vs. calcium containing phosphate binders seem to suggest a survival benefit associated with calcium free phosphate binders. Nonetheless, the numerous limitations of the available studies and the potential publication bias (16) do not currently allow for conclusive recommendations. In addition, in light of the limited resources available and the greater cost burden associated with the use of calcium free phosphate binders, the real cost effectiveness of these compounds need to be addressed by proper pharmacoeconomic analyses, although initial results suggest that Sevelamer may also be cost effective (18).
